# Social network position predicts male mating success in a small passerine

**DOI:** 10.1093/beheco/arab034

**Published:** 2021-05-28

**Authors:** Kristina B Beck, Damien R Farine, Bart Kempenaers

**Affiliations:** 1 Department of Behavioural Ecology and Evolutionary Genetics, Max Planck Institute for Ornithology, Eberhard-Gwinner-Straße, 82319 Seewiesen, Germany; 2 Department of Collective Behavior, Max Planck Institute of Animal Behavior, Universitätsstraße 10, 78464 Konstanz, Germany; 3 Department of Biology, University of Konstanz, Universitätsstraße 10, 78464 Konstanz, Germany; 4 Centre for the Advanced Study of Collective Behaviour, University of Konstanz, Universitätsstraße 10, 78464 Konstanz, Germany; 5 Department of Evolutionary Biology and Environmental Studies, University of Zurich, Winterthurerstrasse 190, 8057 Zurich, Switzerland

**Keywords:** blue tit, extrapair paternity, monogamy, reproduction, social network

## Abstract

Individuals differ in the quantity and quality of their associations with conspecifics. The resulting variation in the positions that individuals occupy within their social environment can affect several aspects of life history, including reproduction. While research increasingly shows how social factors can predict dyadic mating patterns (who will breed with whom), much less is known about how an individual’s social position affects its overall likelihood to acquire mating partner(s). We studied social networks of socially monogamous blue tits (*Cyanistes caeruleus*) to investigate whether the number and strength of connections to opposite-sex conspecifics, the ratio between same- and opposite-sex connections, and the tendency to move between social groups in the months prior to breeding affect individuals’ success in acquiring 1) a breeding partner and 2) an extrapair partner. After controlling for differences in spatial location, we show that males that moved more often between social groups were more likely to acquire a breeding partner. Moreover, adult males that associated with more females were more likely to sire extrapair young. The number of female associates also predicted the proportion of familiar female breeding neighbors, suggesting that familiarity among neighbors may facilitate opportunities for extrapair matings. In females, none of the network metrics significantly predicted the likelihood of acquiring a breeding or extrapair partner. Our study suggests that the positioning of males within their social environment prior to breeding can translate into future mating success, adding an important new dimension to studies of (extrapair) mating behavior.

## Teaser

Differences in individual sociality can have important fitness consequences. Using social network analysis, we test whether differences in individual social position affect the overall likelihood of an individual to acquire mating partners in blue tits. We show that males that moved more often between social groups were more likely to acquire a breeding partner. Further, adult males that associated with more females were more likely to sire extrapair young.

## INTRODUCTION

Within animal groups, individuals typically occupy different social positions (Aplin et al. 2015; Williams et al. 2017; Blaszczyk 2018), which can have fitness-relevant consequences. Differences in individual sociality, characterized by variation in the number and strength of connections to conspecifics and the centrality within the group, have been linked to processes such as the acquisition of information (Aplin et al. 2012; Kulahci and Quinn 2019), the spread of diseases (Godfrey et al. 2009; Hamede et al. 2009), competition for resources (Farine and Sheldon 2015), and survival (Stanton and Mann 2012; Alberts 2019). One dimension of life histories where variation in social position is also important is mating behavior. Here, the number and strengths of connections can ultimately shape reproductive outcomes and sexual selection (Ryder et al. 2009; Oh and Badyaev 2010; Formica et al. 2012; McDonald et al. 2013; McDonald and Pizzari 2018).

An individual’s social position in the group can impact its mating behavior in several ways. For instance, connections to conspecifics are fundamentally linked to mate availability, to the degree of intraspecific competition, and to the potential for sexual harassment (e.g., Jirotkul 1999; Le Galliard et al. 2005; Maldonado-Chaparro et al. 2018; Grant and Grant 2019; Niemelä et al. 2019). Social factors operating at the individual level can generate population-level patterns in terms of which individuals are most likely to breed and with whom they reproduce. For the latter, there is increasing evidence that female–male relationships established prior to breeding can predict dyadic mating patterns (i.e., who will reproduce with whom; Rodway 2007; Psorakis et al. 2012; Teitelbaum et al. 2017; Firth et al. 2018; Maldonado-Chaparro et al. 2018; Beck et al. 2020). Much less is known about whether aspects of an individual’s social position contribute toward determining its overall likelihood to breed.

Studies that examined the relationship between position in the social environment and breeding success often focused on male–male competition. These studies show that not only the focal male’s phenotype but also the composition of the social environment (i.e., the other males’ phenotypes) influence its future success in acquiring a territory (Farine and Sheldon 2015) or in gaining copulations (Formica et al. 2011; Wey et al. 2015; Fisher et al. 2016; Ziv et al. 2016). Furthermore, it has been shown that more central or active individuals (in males: Formica et al. 2012; Sih et al. 2014; in females: Ziv et al. 2016) and those with a higher number of social connections (both sexes: Sabol et al. 2020) gain more copulations. However, many of these studies focused on nonmonogamous mating systems (Formica et al. 2011, 2012; Sih et al. 2014; Wey et al. 2015; Ziv et al. 2016) or examined mating success indirectly through the acquistion of nest sites (Farine and Sheldon 2015). Rarely have studies examined how social factors that relate to the competition for mates contribute to gaining reproductive success in species that form prolonged pair bonds for breeding, that is, socially monogamous species (but see Oh and Badyaev 2010). In monogamous mating systems, the acquisition of a suitable breeding partner is a critical component of an individual’s fitness, particularly for short-lived species where individuals may only have one or a few opportunities to reproduce. Elucidating the (social) factors that determine pairing success in monogamous systems is thus crucial for understanding sources of variation in reproductive success.

Social monogamy with biparental care is the predominant mating system in birds (Black and Hulme 1996). Most species additionally engage in sexual behavior outside their pair bond resulting in extrapair paternity (Brouwer and Griffith 2019). Thus, reproduction can involve two processes: the formation of a social pair bond and the acquisition of extrapair partners. Characteristics that are important for acquiring a social partner may also be relevant for enhancing overall fitness via extrapair offspring. For instance, specific male phenotypic traits can increase both social and extrapair mating success (Thusius et al. 2001, but see Kappes et al. 2009; Manica et al. 2016). In addition to phenotypic characteristics, the social network position could affect the likelihood to acquire a social and extrapair partner. For instance, individuals connected to more conspecifics of the opposite sex and relatively fewer same-sex conspecifics may experience higher mate availability and less competition (Sabol et al. 2020), and thus may be more likely to find social and extrapair mates. Further, individuals moving more frequently between social groups (i.e., that are more central) may have more opportunities to encounter suitable mates (sensu Ihle et al. 2015) and thus may be more likely to acquire a social partner (Oh and Badyaev 2010) and extrapair mates. Social factors may also influence within- and extrapair reproduction in different ways. For example, a high number of associates may allow individuals to find a more preferred social partner (Ihle et al. 2015), and therefore reduce the likelihood of engaging in extrapair copulations. Individuals also differ in how frequently they re-associate with others. Thus, one could predict that individuals with fewer but stronger social bonds should be more likely to find a social partner but less likely to acquire extrapair partners.

In this study, we investigated whether an individual’s social network position prior to breeding predicts its future mating success in a socially monogamous bird, the blue tit (*Cyanistes caeruleus*). During the breeding season, blue tits frequently engage in extrapair matings (Kempenaers et al. 1992; Delhey et al. 2003). In winter, blue tits forage in large mixed-species flocks and female–male dyads with stronger social relationships (i.e., that spent more time foraging together) are more likely to become social or extrapair partners in the subsequent breeding season (Beck et al. 2020). This previous study, conducted in the same population as the present study, therefore confirmed that social associations that occurred prior to the reproductive season predicted who will breed with whom. However, the mechanisms that drive variation in overall mating success (i.e., who will breed at all), and the role of an individuals’ position in the social network therein, remains unclear.

Here, we examined the link between an individual’s overall success in acquiring either a breeding partner or an extrapair partner and four measures of an individual’s social position: 1) the number of opposite-sex associates, 2) the average association strength to the opposite-sex associates, 3) the sex ratio of all its associates, as a measure of intrasexual competition, and 4) the tendency to move between, and therefore connect, different social groups. We predict that individuals that have more opposite-sex connections, that have on average stronger association strengths, that experience less competition (i.e., an opposite-sex biased ratio), and those with a greater tendency to move between social groups will be more likely to acquire a social partner and will be more likely to have had extrapair partners.

## METHODS

### Study species and system

Blue tits are hole-nesting songbirds that form socially monogamous pairs. Males defend a territory during the breeding season but both sexes forage in large mixed-species flocks during winter (Perrins 1979; Farine et al. 2015). In most populations, blue tits only breed once per year, except for some replacement clutches after failure of the first brood. Extrapair paternity occurs in about half of the broods (Kempenaers et al. 1997; Delhey et al. 2003). Extrapair partners are usually close breeding neighbors and adult males are more likely to sire extrapair young than yearling males (Schlicht et al. 2015a).

We collected data from August 2017 until the end of June 2018 in a population located in southern Germany (“Westerholz,” 48°08′26″N 10°53′29″E) that has been studied since 2007. The study site contains 277 nest-boxes that were placed approximately 40 m apart. During the winter (1 November 2017–15 March 2018), we deployed 20 feeders arranged in an even grid across the study site (approximately 200 m apart). All nest-boxes and all feeders were equipped with radio-frequency identification (RFID) antennas (one antenna per nest-box and two per feeder; Loës et al. 2019a, 2019b). During each breeding season, nest-boxes were checked at least once per week (from mid-March onwards) to monitor nest-building activity and to determine laying onset (date of first egg), clutch size and the dates of hatching and fledging.

We trapped birds either at the nest (as nestlings or breeding adults the previous spring) or with mistnets during winter. From every bird, we took a small blood sample (ca. 10 µL) for paternity analysis and molecular sexing. We measured each individual (tarsus length, length of the third primary), weighed it and determined its age based on the color of the wing coverts (yearling (hatched in previous spring) vs. adult (older); Svensson 1992). We also fitted each individual with a numbered metal ring and a uniquely coded passive-integrated transponder (PIT-tag, implanted under the skin on the back). This allowed us to record each visit of a PIT-tagged bird when it came close to the RFID antenna at a nest-box or a feeder (approximately 3 cm). At every detection, the bird’s identity, and the date and time were logged on a SD card. For further details on the study system, see Schlicht et al. (2012).

### Social network

We inferred the social position of individuals by creating a network based on the foraging associations of PIT-tagged birds at feeders. While information about the social behavior of birds is restricted to a foraging context at artificial feeders and thus may not represent their natural association patterns, previous work on tits (Paridae) has demonstrated that foraging associations are meaningful in predicting processes in other contexts such as the discovery of novel food patches (Aplin et al. 2012; Hillemann et al. 2020), the spatial breeding arrangement (Firth and Sheldon 2016; Beck et al. 2020) and mating patterns (Beck et al. 2020). We used data from the two months before the start of breeding (from 1 February until 13 March 2018) to create a social network. We chose this time window, because dyadic foraging associations during this period predicted both future social and extrapair partners (i.e., who bred with whom), in contrast to foraging associations earlier in winter (Beck et al. 2020). Thus, associations during late winter (i.e., February and March) might be most important for future mating outcomes.

We defined an “association” as two birds foraging together within the same flock. We assigned individuals to flocking events using Gaussian Mixture Models (Psorakis et al. 2012, 2015) with the function “gmmevents” from the R (R Development Core Team 2018) package “asnipe” (Farine 2013). The temporal pattern of recorded visits will typically contain periods of high activity separated by periods of no activity (as birds forage in flocks, see Supplementary Figure S1). The “gmmevents” function allows us to detect these events of increased feeding activity in the spatio-temporal data and clusters these events into nonoverlapping gathering events (i.e., flocking events), without using arbitrary temporal boundaries to define a flock. Individual visits were then assigned to the corresponding flocking event, allowing us to see which individuals co-occurred in the same flock. For more detailed information, see Supplementary Figure S1 and Psorakis et al. (2012, 2015).

We inferred the strength of associations among individuals from the patterns of co-occurrences in flocks. We calculated association strength using the simple ratio index (SRI) because the data on associations are incomplete (i.e., we could not observe associations that took place away from the feeder; Cairns and Schwager 1987; Hoppitt and Farine 2018). The SRI describes the proportion of observations of two individuals in which they were seen together, thus ranging from 0 (never observed in the same flock) to 1 (always observed in the same flock). We created an undirected network with edges weighted by the SRI from the whole study period (1 February–13 March). We then derived for each individual the number of opposite-sex associates (i.e., the degree), the average association strength to the opposite-sex associates (i.e., the average of an individual’s edge weights), the sex ratio (i.e., the number of same-sex associates divided by the total number of associates), and the unweighted betweenness centrality (Freeman et al. 1979) using the R package “igraph” (Csardi and Nepusz 2006). The latter represents the number of shortest paths between individuals that pass through the focal individual. Betweenness centrality thus reflects to what extent an individual connects disparate parts of a network and represents an individual’s tendency to move between different groups (Farine and Whitehead 2015). We calculated these four metrics based on all observed associations since it is generally advised against thresholding networks (i.e., the removal of weak edges; Langer et al. 2013; Farine 2014; Farine and Whitehead 2015) and also fleeting associations might be important for mating outcomes. However, we additionally repeated our main analyses removing 5% and 10% of weakest edges (see Supplementary Tables S1–S8). We chose these four metrics because previous studies showed that they influence mating in other systems (e.g., number of associates: Sabol et al. 2020, betweenness centrality: Oh and Badyaev 2010, sex ratio: Grant and Grant 2019) and because we can make meaningful predictions of how they may influence mating outcomes in blue tits (see Introduction).

### Pairing success

We defined a bird as having successfully acquired a social partner if we detected it breeding in one of the nest-boxes in our study site. We quantified breeding pairs based on the PIT-tag detections at nest-boxes throughout the breeding season. Both pair members visit their breeding box frequently from nest-building onwards until their young fledge. We defined individuals as having been unsuccessful in acquiring a social partner if they were still present in the study site (i.e., detected at least once at one or more nest-boxes) during the breeding season but did not breed in any of the boxes. We defined the start of the breeding season as the day on which the first nest material was found inside a nest-box (14 March). We cannot exclude the possibility that “unsuccessful” individuals bred in natural cavities within or outside our study site. However, we suspect that the number of such birds within the study site is small, because we provided an excess of nest-boxes (i.e., high-quality nest sites). In 2018, only 135 nest-boxes (48.7%) were occupied. Further, since 2007, we only recorded a single pair breeding in a natural cavity within our study site, but we may have missed other cases.

### Extrapair paternity

We genotyped nestlings and adults using 14 microsatellite markers and one sex chromosome linked marker (Supplementary Table S9). Microsatellite amplifications were performed in multiplexed PCRs and primer mixes containing two to five primer pairs (Supplementary Table S9). We compared the genotypes of parents and their offspring using the software CERVUS (Kalinowski et al. 2007). No cases of intraspecific brood parasitism were recorded, that is, the social female at a nest-box was always assigned the genetic mother. We determined whether a brood contained extrapair young and assigned the genetic father to the majority (90.5%) of these extrapair young. For further information on the paternity analysis, see Delhey et al. (2003) and Schlicht et al. (2012).

### Statistical analyses

#### Pairing success

We examined the effect of individuals’ social network position on their pairing success by fitting generalized linear models (GLMs) in R (R Development Core Team 2018). Analyses were performed separately for males and females and only included data from birds that were present during winter and breeding, and that had been equipped with a transponder before the start of the study (1 February 2018). Further, we excluded birds that had bred in our study site in previous years to exclude effects of previous breeding experience (remaining sample size: *N*_Males_ = 119, *N*_Females_ = 95) and repeated the analyses excluding all adult birds (remaining sample size: *N*_Males_ = 75, *N*_Females_ = 67). We included as dependent variable whether the individual bred in 2018 or not (“binomial error structure”) and as explanatory variables the four social network measures: 1) the number of opposite-sex associates, 2) the average association strength to opposite-sex associates, 3) the sex ratio, and 4) the betweenness centrality. Further, we included each individual’s arrival date at the study site, because this also affects the likelihood to breed (Gilsenan et al. 2020), and their age (yearling vs. adult), because adult individuals might be more likely to breed. We defined arrival date as the first day of the season an individual was recorded at a nest-box or feeder (starting 1 August 2017, following Gilsenan et al. 2020). We standardized each variable by subtracting the mean and dividing by two times the standard deviation, using the “standardize” function of the R package “arm” (Gelman 2008; Gelman and Su 2018). Correlation coefficients among all fixed effects were below the suggested threshold (*r* < 0.5–0.7, Supplementary Table S10) by Dormann et al. (2013) to reach correct model estimations.

#### Extrapair paternity

We examined the effect of an individuals’ social network position on extrapair mating by fitting GLMs. We analyzed the data separately for males and females and only included data from birds that had been equipped with a transponder before the start of the study and that were present during winter. For females, we only included individuals that bred in the subsequent breeding season (*N* = 95). For males, we included all individuals present during the breeding season (i.e., detected at least once at one or more nest-boxes) regardless of whether they bred in one of our nest-boxes (*N* = 123). In addition, we repeated the analysis only including males that bred (*N* = 81). We included as dependent variable whether the individual had extrapair young or not (“binomial error structure”) and as explanatory variables the four social network measures as described above in the section on pairing success. For males, we only included adults (i.e., older than one year) as only few yearlings sired extrapair young (Schlicht and Kempenaers 2013) (in 2018: only four). We standardized all explanatory variables and checked for correlations among the fixed effects (all *r* < 0.5, Supplementary Table S11) as described in the section on pairing success.

#### Null models

Social networks are based on nonindependent data of multiple individuals, violating the assumptions of many statistical tests (Croft et al. 2011). Thus, we used node permutations (Croft et al. 2008; Whitehead 2008) to account for the nonindependence in our data and to determine the effect of the social network measures on the likelihood to acquire a breeding partner or an extrapair partner. In node permutations, the identity of each node is randomized, breaking the link between the social network metrics and individual identities, while the link between individual identity and other individual-level predictors such as age and arrival date is maintained (Croft et al. 2008; Whitehead 2008). We first performed a spatially unrestricted permutation by randomly swapping the network position of same-sex individuals. Second, we performed a spatially restricted node permutation. This location-specific null model allowed us to control for potential confounding effects that would influence our social network metrics if individuals were nonrandomly distributed in space, thereby driving spurious correlations with the dependent variable. This approach also enabled us to partially differentiate between patterns arising from social preferences versus those arising from spatial decisions. For instance, certain habitat configurations (e.g., vegetation, density, presence of predators, etc.) in the location where an individual preferably forages may influence the social network metrics. In such a case, individual differences in network metrics may not necessarily arise from differences in social behavior but from differences in habitat preferences. We determined each individual’s preferred feeder as the one it most often visited. Ideally, we would have swapped the network positions of those same-sex individuals that preferably foraged at the same feeder. However, as some feeders were only preferred by few individuals (six feeders with fewer than three individuals), randomizations within each feeder location would not be meaningful. Thus, we clumped the feeders into spatial clusters, each containing at least 10 individuals of each sex. This resulted in five distinct clusters (each comprising three to five feeders, Supplementary Figure S2), and allowed us to restrict the randomizations to individuals of the same sex and from the same spatial cluster.

We repeated the node permutations for the spatially unrestricted and the location-specific null model 1000 times. After each permutation, we repeated the GLM as described above in the sections on pairing success and extrapair paternity, and compared the coefficient of the slope of the four network metrics from the observed data to the distribution of coefficients from 1000 models fitted to the randomized data. Cases where the observed value lays outside the 95% range of the distribution of randomized values indicated a statistically significant effect. If the observed data differ from the location-specific null model, differences in the network metrics and their potential effect on mating success are likely caused by differences in social behavior rather than spatial effects.

#### Post-hoc analyses

We found that males that associated with more females during winter were more likely to sire extrapair young (see Results). Because individuals breeding in neighborhoods with higher densities are usually more likely to have extrapair young (Schlicht et al. 2015a), we examined whether the number of female associates predicted 1) the number of neighbors and 2) the proportion of familiar females (familiarity defined as having associated during winter) within the close neighborhood in the subsequent breeding season. To characterize an individual’s breeding neighborhood, we assigned territories to breeding pairs based on Thiessen polygons, using the R package “expp” (Valcu and Schlicht 2013). Based on this information, we defined for each focal pair that bred in the study site the first-order neighbors as those that shared a territory border, and the second-order neighbors as those that have one territory in between them (for further details, see Valcu and Schlicht 2013; Schlicht et al. 2015a). We defined the close neighborhood as those including first- and second-order neighbors because most extrapair sires belong to this neighborhood (Schlicht et al. 2015a; 51% and 32% of extrapair sires were first- and second-order neighbors, respectively). We repeated the analyses only including direct neighbors (first order). We fitted GLMs and included as dependent variable 1) the number of direct neighbors in the close neighborhood (“Poisson error structure”) and 2) the proportion of familiar female neighbors, fitted as a matrix including the number of familiar neighbors and the number of unfamiliar neighbors (“binomial error structure”). As explanatory variable, we included the number of female associates during winter. We examined the effect of the number of female associates by performing spatially restricted and unrestricted node permutations as described above.

In addition, we examined whether the number of female associates could be one of the underlying reasons for the increased extrapair siring success of adult compared to yearling blue tits (Schlicht and Kempenaers 2013). We compared the number of female associates between adult and yearling males using a Wilcoxon rank sum test.

## RESULTS

Between 1 February and 13 March 2018, we recorded 13,095 flocking events at feeders (on average 19 per feeder per day), comprising 452 individuals (242 males and 210 females). Individuals were recorded on average 24 days (SD = 15.1, range: 1–48) and used six different feeder locations (SD = 3.4, range: 1–17). Of all recorded individuals, 221 (48.9%) were recorded breeding in a nest-box.

### Pairing success

**Figure 1:**
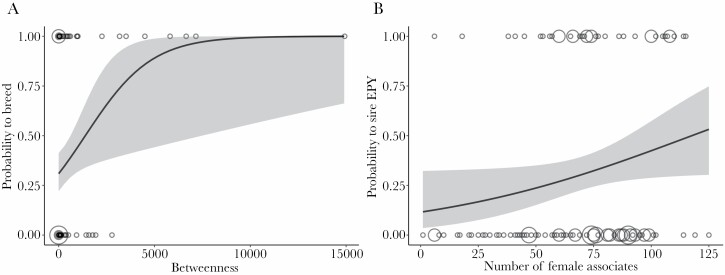
Mating success of male blue tits in relation to their winter network position. (A) The predicted probability that a male formed a social pair and bred in relation to the betweenness centrality. (B) The predicted probability that an adult male sired extrapair young (EPY) in relation to the number of female associates. Dots show the raw data and dot size represents the number of individuals (A: *N* = 1–30, B: *N* = 1–3). The grey ribbon shows the 95% confidence interval from the generalized linear model described in the main text while keeping all other independent variables constant at their mean values (standardized effects are shown in Table 1 and 2). Note that the effect was still present after excluding the outlier in betweenness centrality in (A) (Supplementary Table S14, Supplementary Figure S7).

The analysis of pairing success included 119 males (46 successfully paired, 73 did not pair) and 93 females (41 successfully paired, 52 did not pair). Yearling males, males that arrived earlier, and those with a larger betweenness centrality (i.e., a greater tendency to move between different flocks) were more likely to breed in the subsequent spring (mean betweenness centrality ± SD: breeding individuals: 1180.5 ± 2748.2; not breeding: 180.2 ± 504.4; Figure 1A, Supplementary Figures S3A and S4A, Table 1). The betweenness centrality of males ranged from 0 to 14923, and for every 100 unit increase in the betweenness centrality the odds of a male to breed increased on average by 1.06 times. A juvenile male with an average betweenness centrality of 566.7 had a 47% probability to acquire a breeding partner, while an adult male with the same betweenness centrality only had a 25% probability to breed (while keeping all other independent variables at their mean values; Figure 1A). Arrival date ranged from 3 to 223 and for every day later arrival the odds of a male breeding decreased by 0.99 times (Supplementary Figure S5). The other network metrics did not predict the likelihood of breeding (Supplementary Figure S5, Table 1).

**Table 1:** Results of two models examining the effect of the number of opposite-sex associates, the average association strength, the sex ratio, the betweenness centrality, age (yearling vs. adult) and arrival date on the likelihood to acquire a social partner and breed for males (*N* = 119) and females (*N* = 93). Significant *P* values are shown in bold. *P* values inferred from 1000 random permutations are shown in italic (∆: spatially unrestricted null model, *: location-specific null model)

Fixed effect	Males			Females		
	Estimate ± SE	z	P	Estimate ± SE	z	P
Intercept	−0.48 ± 0.22	−2.14		−0.29 ± 0.24	−1.23	
Number of associates	−0.37 ± 0.50	−0.74	0.44 ^∆^	−0.35 ± 0.61	−0. 58	0.52 ^∆^
			0.41 *			0.51 *
Average association strength	0.28 ± 0.48	0.58	0.53 ^∆^	−0.28 ± 0.51	−0.54	0.60 ^∆^
			0.55 *			0.59 *
Sex ratio	−0.20 ± 0.47	−0.43	0.66 ^∆^	0.59 ± 0.52	1. 14	0.26 ^∆^
			0.66 *			0.29 *
Betweenness centrality	**2.10 ± 0.91**	**2.32**	**0.01** ^**∆**^	0.70 ± 0.61	1. 15	0.27 ^∆^
			0.02 *			0.26 *
Age^a^	−**1.00 ± 0.47**	−**2.13**	**0.03**	−**1.68 ± 0.60**	−**2.82**	**0.01**
Arrival date	−**1.48 ± 0.49**	−**3.04**	**0.002**	−1.08 ± 0.57	−1.89	0.06

^a^Adults compared to yearlings.

The effect of betweenness centrality on pairing success remained unchanged when controlling for spatial location (Supplementary Figure S3A, Table 1). We repeated the analysis only including yearling males (*N* = 75) and while the effect of arrival date was still present, the effect of betweenness centrality was no longer statistically significant, but still positive (Supplementary Table S12). In females, none of the network metrics predicted the likelihood of social pairing success, but yearlings were also more likely to breed than adults (Table 1). Repeating the analysis with only yearling females (*N* = 67) showed that earlier arriving individuals were more likely to breed (Supplementary Table S12).

### Extrapair paternity

The analysis on extrapair paternity included 123 adult males (excluding 75 yearlings) and 95 females. Of those, 38 adult males sired extrapair young and 36 females had extrapair young in their brood. Males that associated with more females during winter were more likely to sire extrapair young, even when controlling for spatial location (mean number of female associates ± SD: extrapair sires: 75.0 ± 26.5; males that did not sire extrapair young: 64.7 ± 28.7; Figure 1B, Supplementary Figures S3B and S4B, Table 2). The number of female associates ranged from 2 to 125. One unit increase in the number of female associates led to a 1.02 time increase in the odds of an adult male to sire extrapair young. An adult male with an average number of 67.9 female associates had a 30% probability to acquire an extrapair partner with whom he sired at least one offspring (while keeping all other independent variables constant at their mean values, Figure 1B). The other network metrics did not predict the occurrence of extrapair paternity (Supplementary Figure S6, Table 2). The number of female associates did not differ between adult and yearling males (mean ± SD (range); adults: 67.9 ± 29 (1–125), yearlings: 73.8 ± 27 (4–142), Wilcoxon rank sum test: W = 5157, *P* = 0.16, inferred from 1000 random permutations). The results remained the same when only including males that had been detected breeding (81 adult males of which 32 sired extrapair young, Supplementary Table S13). Further, among those males that bred, a higher number of female associates did not translate into having more neighbors during breeding (Table 3), but into a greater proportion of familiar females within the close breeding neighborhood (Table 3). Results remained the same when only considering direct neighbors (data not shown). In females, none of the examined social network metrics predicted the occurrence of extrapair paternity in their brood, although the effect of the number of male associates was also positive (Table 2).

**Table 2:** Results of two models examining the effect of the number of opposite-sex associates, the average association strength, the sex ratio, and the betweenness centrality on the likelihood to acquire extrapair young for adult males (*N* = 123) and for females (*N* = 95). Significant *P* values are shown in bold. *P* values are inferred from 1000 random permutations (∆: spatially unrestricted null model, *: location-specific null model)

Fixed effect	Males			Females		
	Estimate ± SE	z	*P*	Estimate ± SE	z	*P*
Intercept	−0.86 ± 0.21	−1.20		−0.55 ± 0.23	−2.45	
Number of associates	**0.98 ± 0.49**	**2.01**	**0.03** ^**∆**^	0.93 ± 0.53	1. 74	0.08 ^∆^
			0.04 *			0.07 *
Average association strength	0.48 ± 0.43	1.12	0.27 ^∆^	−0.02 ± 0.46	− 0.05	0.96 ^∆^
			0.27 *			0.96 *
Sex ratio	0.40 ± 0.46	0.43	0.38 ^∆^	0.91 ± 0.50	1. 82	0.07 ^∆^
			0.41 *			0.08 *
Betweenness centrality	−0.03 ± 0.39	−0.08	0.94 ^∆^	−1.14 ± 0.78	− 1.47	0.10 ^∆^
			0.93 *			0.10 *

**Table 3:** Results of models examining the effect of the number of female associates during winter on the number and on the proportion of familiar females in the close breeding neighborhood (first- and second-order neighbors) of males (*N* = 81). Significant P values are shown in bold. *P* values are inferred from 1000 random permutations (∆: spatially unrestricted null model, *: location-specific null model)

	Number of neighbors			Proportion of familiar females		
	Estimate ± SE	z	*P*	Estimate ± SE	z	*P*
Intercept	2.59 ± 0.03	84.65		1.03 ± 0.07	14.13	
Number female associates	0.09 ± 0.06	1.42	0.19 ^∆^	**1.19 ± 0.14**	**8.42**	**<0.001 ** ^∆^
			0.77 *			<0.001 *

## DISCUSSION

Recent studies have shown that animal social structure can affect various ecological processes and fitness outcomes (Croft et al. 2016; Webber and Vander Wal 2019; Cantor et al. 2020). Although the link between the social environment and mating behavior has received much attention, few studies examined how social factors contribute to gaining reproductive success in socially monogamous species. Here, we demonstrate that the social position of a male blue tit during winter has consequences for its success in acquiring both a breeding partner and extrapair partners. Males with a greater tendency to move between flocks (a higher betweenness centrality) were more likely to form a pair and breed than males that moved less. Further, adult males that were connected to more females during winter were more likely to sire extrapair young in the subsequent breeding season. Social network metrics did not significantly predict the probability of breeding or of having extrapair offspring in females, which indicates that social or sexual selection (e.g., through female-female competition) is less strong in females than in males.

Individuals can actively modify their social environment to increase mating success (Jirotkul 2000; Oh and Badyaev 2010; Formica et al. 2011). For instance, in house finches (*Carpodacus mexicanus*), males with a less elaborate plumage changed social groups more frequently (i.e., expressed a higher betweenness centrality) compared to more elaborately colored males (Oh and Badyaev 2010). This increased the relative attractiveness of less ornamented individuals, leading to an increased pairing success (Oh and Badyaev 2010). In our study, focused on males without previous breeding experience, we found that individuals that moved more frequently between flocks during the winter were subsequently more likely to be observed breeding (Figure 1A, Table 1). A higher betweenness centrality might reflect a higher probability for an individual to find a suitable social partner or an increased likelihood to acquire a territory. When analyzing only yearling males the effect was still positive but no longer statistically significant (Supplementary Table S12), presumably due to lower statistical power. The process(es) behind the effect of this social metric and whether this differs between yearlings and adults warrant further exploration.

Variation in extrapair paternity has often been linked to characteristics of the breeding environment such as the breeding density (Westneat and Sherman 1997; Thusius et al. 2001; Schlicht et al. 2015a) or synchrony (Stutchbury and Morton 1995; Chuang et al. 1999; Thusius et al. 2001). However, recent evidence suggests that extrapair paternity does not only arise from conditions during breeding but could be linked to pre-breeding associations between females and males (Maldonado-Chaparro et al. 2018; Beck et al. 2020). A study on the same blue tit population showed that female–male dyads with stronger associations during winter were more likely to become extrapair partners (Beck et al. 2020). Thus, social connectivity prior to breeding seems to be important for future mating success. This raises the question whether not only the quality of dyadic associations but also an individual’s overall social network position contributes to its future extrapair siring success. Here, we report that a male’s success in siring extrapair young increased when it associated with more females prior to breeding (Figure 1B, Table 2). Thus, the social environment in winter influences both which male–female dyads become extrapair partners (Beck et al. 2020) and a male’s likelihood to sire extrapair young (for adults; this study).

Our finding then raises the question how more connections to females during winter translate into an increased likelihood to sire extrapair young? One possibility is that more associates during winter lead to a higher local breeding density, which may in turn result in more potential extrapair partners. However, we found no such effect (Table 3). Instead, we show that having more female associates in winter translated into a higher proportion of familiar females within the close breeding neighborhood (Table 3). Visits of male blue tits to neighboring territories are associated with a higher likelihood to sire extrapair young with the female of the visited nest-box (Schlicht et al. 2015b). Familiarity among breeding neighbors may facilitate such visits and hence extrapair copulations, for instance if it reduces aggression or if it increases the likelihood that a female accepts a copulation attempt. Beck et al. (2020) also suggested that familiarity between female–male dyads from foraging together in winter led to an increased likelihood of becoming extrapair partners. However, how familiarity among neighbors facilitates extrapair copulations needs further investigation. Yearling males are much less likely to sire extrapair offspring than adults, despite having an equal number of female associates compared to adult males. For males, the number of females with whom they had a connection was strongly correlated with the number of male associates (*r* = 0.94) and the sex ratio had no effect on extrapair siring success (Table 2). Therefore, it seems unlikely that the increased success in siring extrapair young is caused by reduced male–male competition. However, we inferred the social environment from an artificial foraging context which may have led to an unusual high density of birds. Thus, data on natural associations would be beneficial.

Our findings show that the features of the winter social environment that predict a male’s success in acquiring a social and an extrapair partner differ (betweenness centrality versus number of female associates). Thus, the acquisition of a social partner may be based on a different underlying process than the acquisition of extrapair partners. Beck et al. (2020) suggested that the association with the social partner develops earlier in winter than associations with future extrapair partners. For social partners, it might be beneficial to bond early to synchronize their behavior (Spoon et al. 2006; Griggio and Hoi 2011; Ihle et al. 2015). Moving more frequently between social groups may facilitate finding a suitable (or available) breeding partner. In contrast, siring extrapair young probably does not require a prolonged pair formation process during winter, but may simply arise from opportunities to engage in extrapair copulations (Beck et al. 2020). We speculate that such opportunities increase if more familiar females are present in the breeding neighborhood (see above).

Individual differences in social network metrics do not necessarily result from differences in social behavior but may be affected by other factors such as spatial effects (He et al. 2019; Albery et al. 2020). For instance, if individuals prefer to forage at sites with denser vegetation (e.g., because of reduced risk of predation), local density at such sites may be higher, which then leads to a higher number of social associates compared to individuals that forage at lower-density sites. However, in our study, controlling for the potential effect of spatial location, by randomizing individuals foraging in the same spatial cluster (Supplementary Figure S2), did not affect the conclusions (Tables 1 and 2). This outcome suggests that the effects of an individual’s social network position are not simply due to spatial effects. However, the differences between the observed effect sizes and those generated by the location-specific null model were smaller than the differences with the spatially unrestricted null model (compare the ∆ values in Supplementary Figure S3) which suggests that the spatial location does also contribute to the observed effect.

The relationship between mating success and social network position may also result from underlying phenotypic traits that themselves influence both mating success as well as network position. For instance, in the closely related great tit (*Parus major*), differences in personality have been linked to variation in extrapair paternity (Van Oers et al. 2008) and to differences in social position (Aplin et al. 2013). Thus, before concluding that social behavior itself is the target of selection, we need to determine that the link between social network position and mating success is not due to other underlying phenotypic traits. The link between extrapair siring success and the number of prior associates could for example arise if females preferably associate with higher-quality or dominant males. Several male traits have been linked with extrapair mating success in blue tits (e.g., body size: Schlicht et al. 2015a; plumage coloration: Delhey 2006, but see Delhey et al. 2003; song characteristics: Kempenaers et al. 1997). Further work is now needed to determine the male traits underlying individual social network position, and to better understand how social network position translates into mating success.

## FUNDING

This work was supported by the Max Planck Society. D.R.F. received additional funding from the Deutsche Forschungsgemeinschaft (DFG grant FA 1402/4-1), the DFG Centre of Excellence 2117 “Centre for the Advanced Study of Collective Behaviour” under Germany’s Excellence Strategy—EXC 2117—422037984, the European Research Council (ERC) under the European Union’s Horizon 2020 research and innovation programme (grant agreement No. 850859), and an Eccellenza Professorship Grant of the Swiss National Science Foundation (Grant Number PCEFP3_187058).

We thank all members of the field team, and especially Agnes Türk, Andrea Wittenzellner, Carles Durà, Cécile Vansteenberghe, Friederike Böhm, and Giulia Bambini for help with data collection, and Peter Loës and Peter Skripsky for nest-box and feeder maintenance. We are grateful to Sylvia Kuhn and Alexander Girg for microsatellite genotyping, and to Mihai Valcu for comments on the analyses. We thank the Bavarian regional office for forestry (LWF) for permission to work in Westerholz. We thank Amanda Ridley and two anonymous reviewers for constructive comments on the manuscript.

## AUTHOR CONTRIBUTIONS

All authors conceived the idea and designed the study. B.K. conducted the paternity analyses; K.B. analyzed the data with input from D.F. and B.K.; K.B., D.F., and B.K. wrote the paper.

## Data availability

Analyses reported in this article can be reproduced using the data provided by Beck et al. 2021.

## Supplementary Material

arab034_suppl_Supplementary_Material
