# Supplementary material for: Social network position predicts male mating success in a small passerine
Source: Behav Ecol. 2021 May 28;32(5):856–64. doi: 10.1093/beheco/arab034 (PMC8528538; doi:10.1093/beheco/arab034)
Supplement: arab034_suppl_Supplementary_Material [file arab034_suppl_supplementary_material.docx]

**Supplementary material for:**

**Social network position predicts male mating success in a small passerine**

Kristina B. Beck^1^ , Damien R. Farine^2,3,4,5^, Bart Kempenaers^1^

^1^ Department of Behavioural Ecology and Evolutionary Genetics, Max Planck Institute for Ornithology, Seewiesen, Germany

^2^ Department of Collective Behavior, Max Planck Institute of Animal Behavior, Konstanz, Germany

^3^ Department of Biology, University of Konstanz, Germany

^4^ Centre for the Advanced Study of Collective Behaviour, University of Konstanz, Germany

^5^ Department of Evolutionary Biology and Environmental Studies, University of Zurich, Switzerland

**Additional figures**


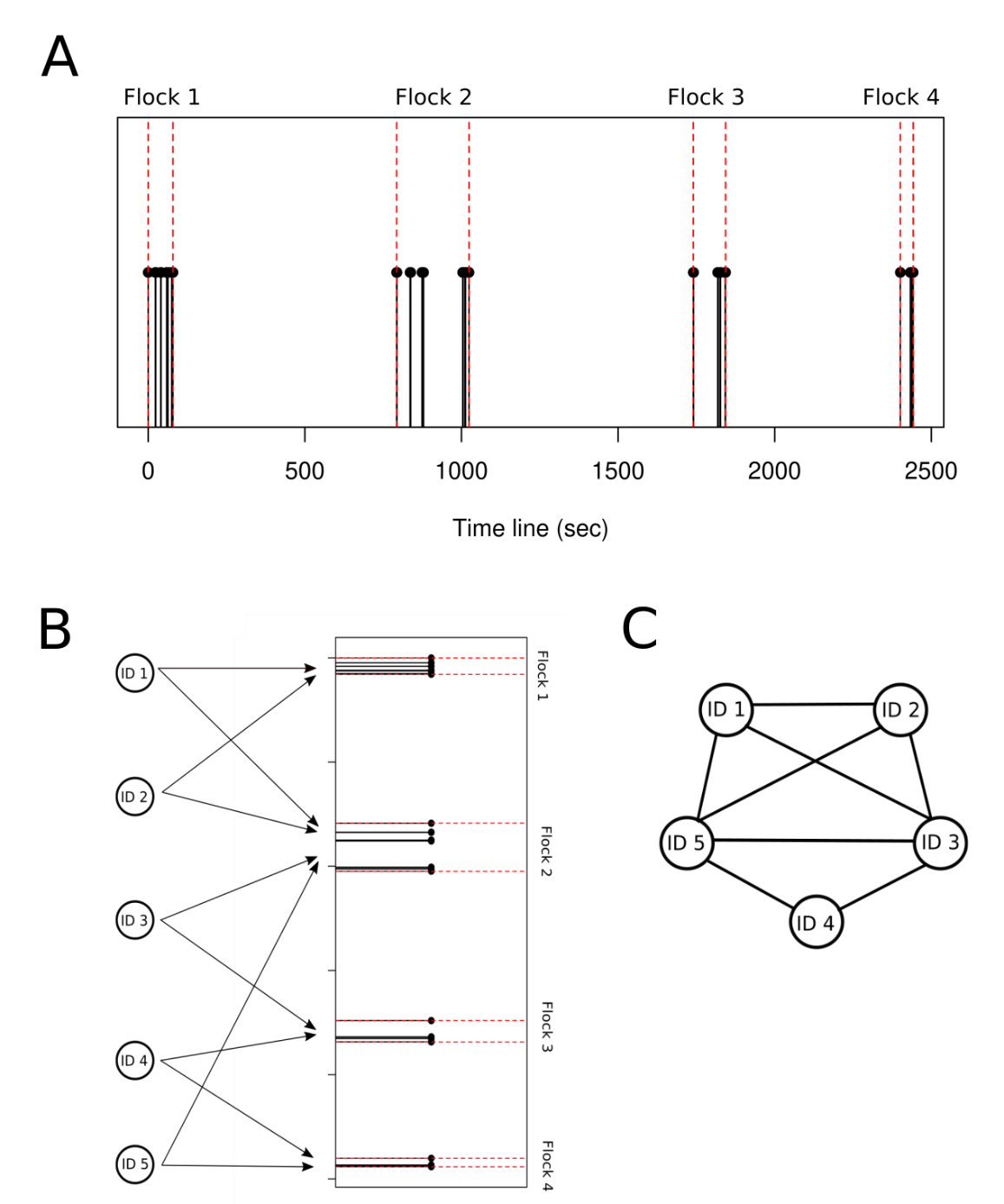


**Figure S1.** Schematic representation of the method to assign individuals to a particular foraging flock. **A)** Data stream showing the arrival of birds (black dots and lines) to a feeder over time. Visits of birds are clumped and assigned into “flocking” events. For each identified flocking event, the start and end are indicated by a left and right red, dashed line. **B)** Individuals are assigned to the identified flocking events in a bipartite network. **C)** Social networks are created based on the co-occurrence of birds within the same flocking events. Figure adapted from Figure 2 and Figure 4 in Psorakis et al. 2012.

**
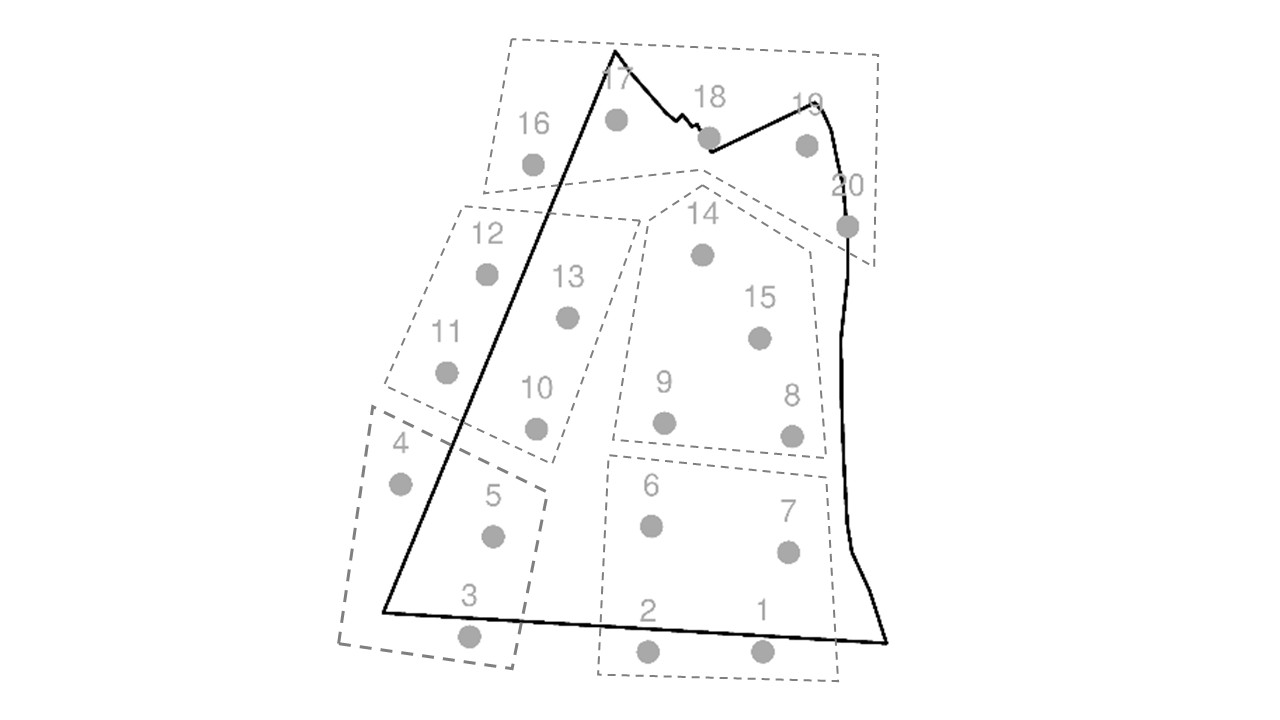
**

**Figure S2.** Sketch of our study site. The black outline represents the boundary of the forest patch (approximately 40 ha) including all breeding nest boxes. The dashed grey outline represents the spatial clusters of feeders (grey dots).


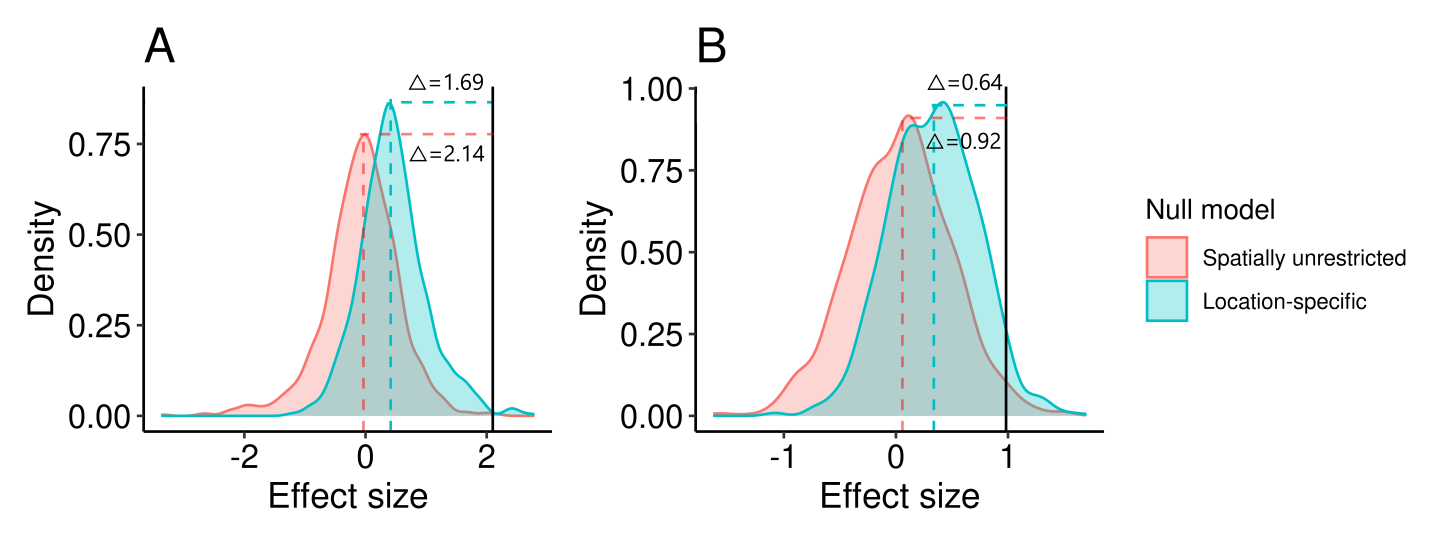


**Figure S3.** Distribution of the effect sizes of the observed and randomized data for males. (**A**) Effect sizes of the betweenness centrality on social pairing success. (**B**) Effect sizes of the number of female associates on extra-pair siring success. The black line indicates the effect size of the observed data (see also Table 1 and 2). The density plots show the distribution of effect sizes generated with the spatially unrestricted null model (red) and the location-specific null model (blue). The median effect size for each density plot is shown as a vertical dashed line in the corresponding colour. Shown is also the difference (△) between the observed effect size and the median effect size of the spatially unrestricted null model and the location-specific null model.


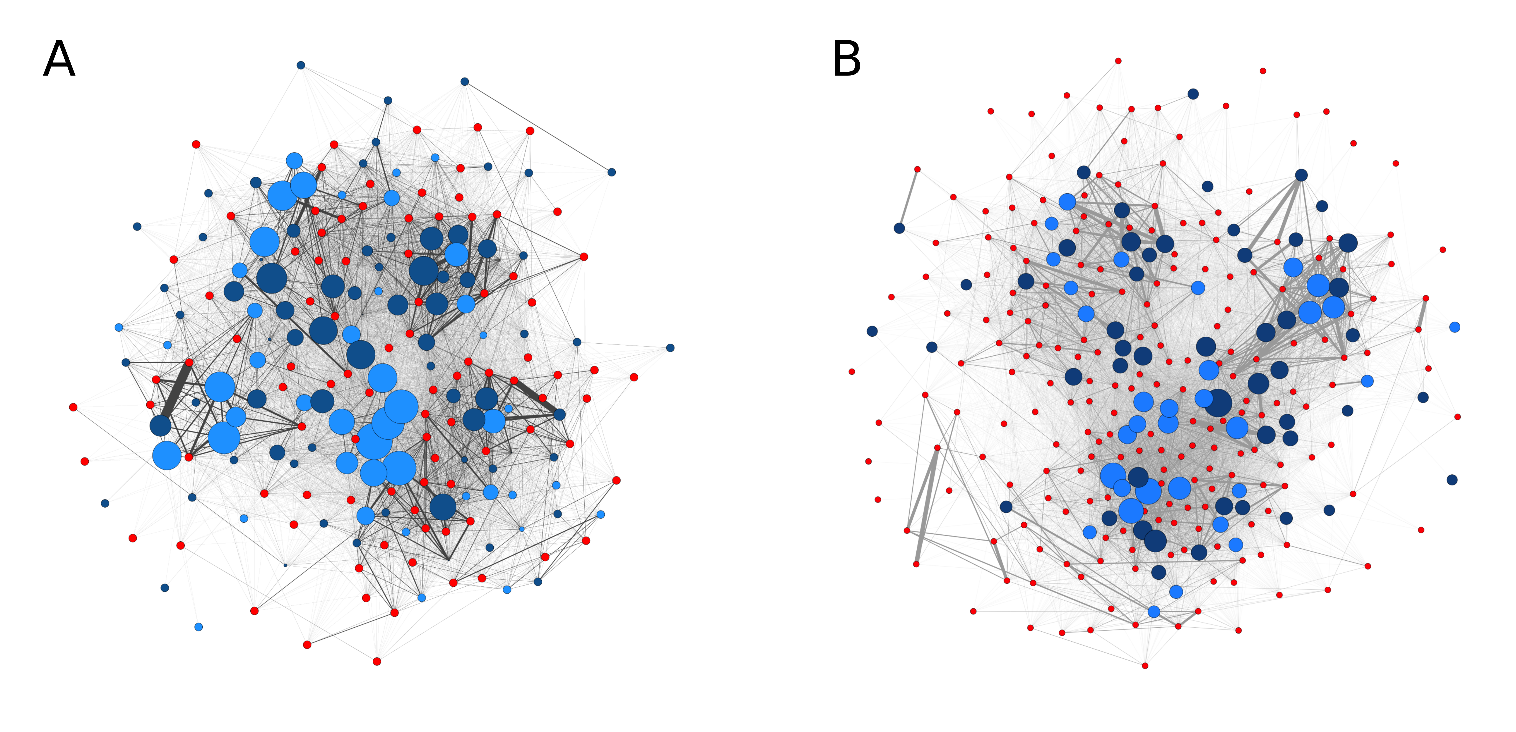


**Figure S4.** (**A**) Social network representing all males included in the analysis on pairing success (N=119) and their connections to females and males during the study period. Females are represented by red nodes, males that successfully acquired a breeding partners in light blue and “unsuccessful” males in dark blue. Node size in males represents their betweenness centrality (i.e. larger nodes indicate more central males); node size in females is kept constant. (**B**): Social network representing all males included in the analysis on extra-pair siring success (N=81) and their connections to females. Light blue indicates males that sired extra-pair young, dark blue shows males that did not. Node size in males represents their degree (i.e. larger nodes indicate a higher number of female connections); node size in females (red) is kept constant. The thickness of lines between nodes in plot A and B represents the edge weight (i.e. thicker lines indicate stronger associations).


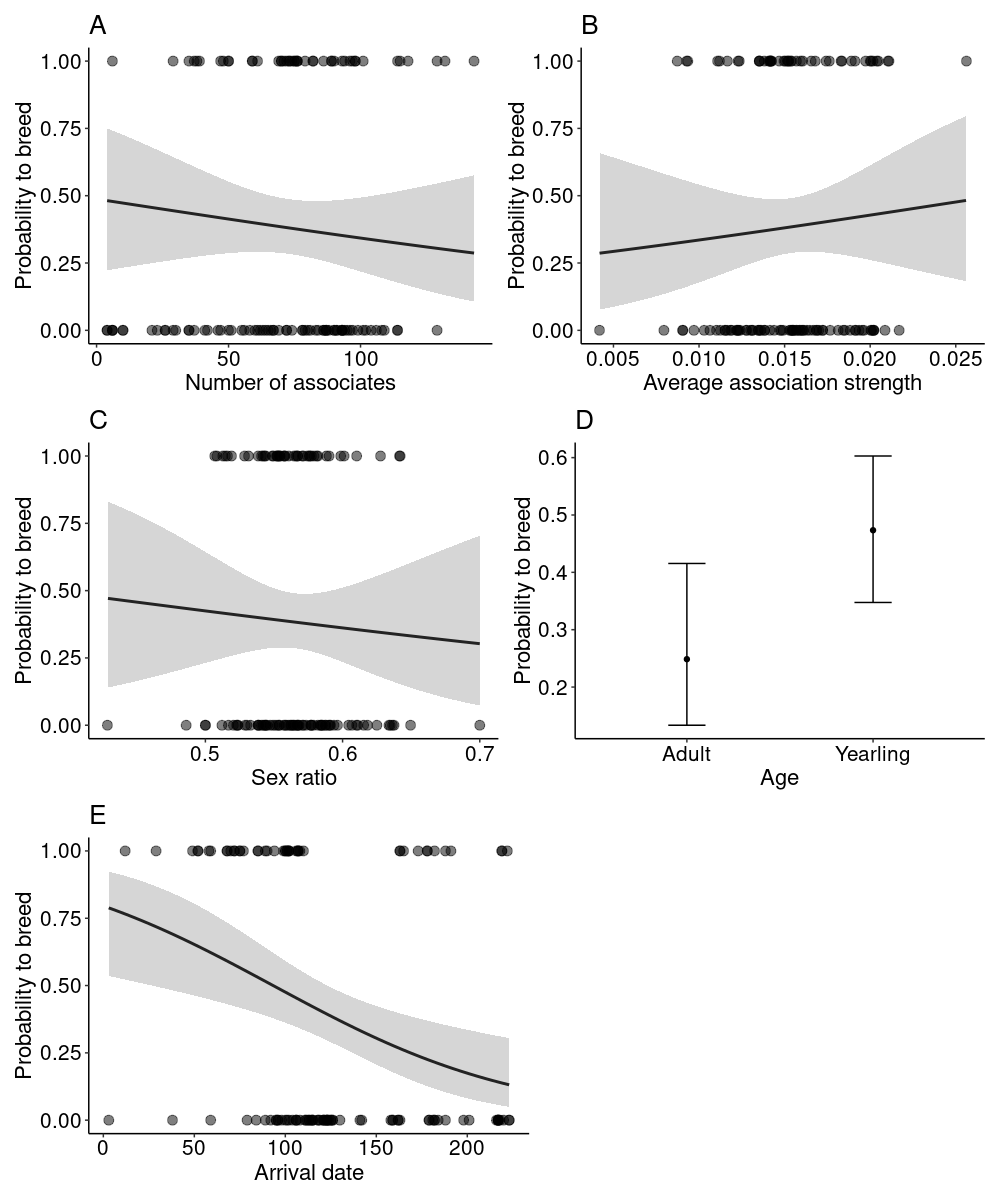


**Figure S5.** Correlates of pairing success of male blue tits. Shown is the predicted probability that a male acquired a breeding partner in relation to (A) the number of female associates, (B) average association strength, (C) sex ratio, (D) age and (E) arrival date. Dots show the raw data and the grey ribbon (A,B,C,E) or the error bars (D) show the 95% confidence interval from the generalized linear model described in the main text, while keeping all other independent variables constant at their mean values (standardized effects are shown in Table 1).


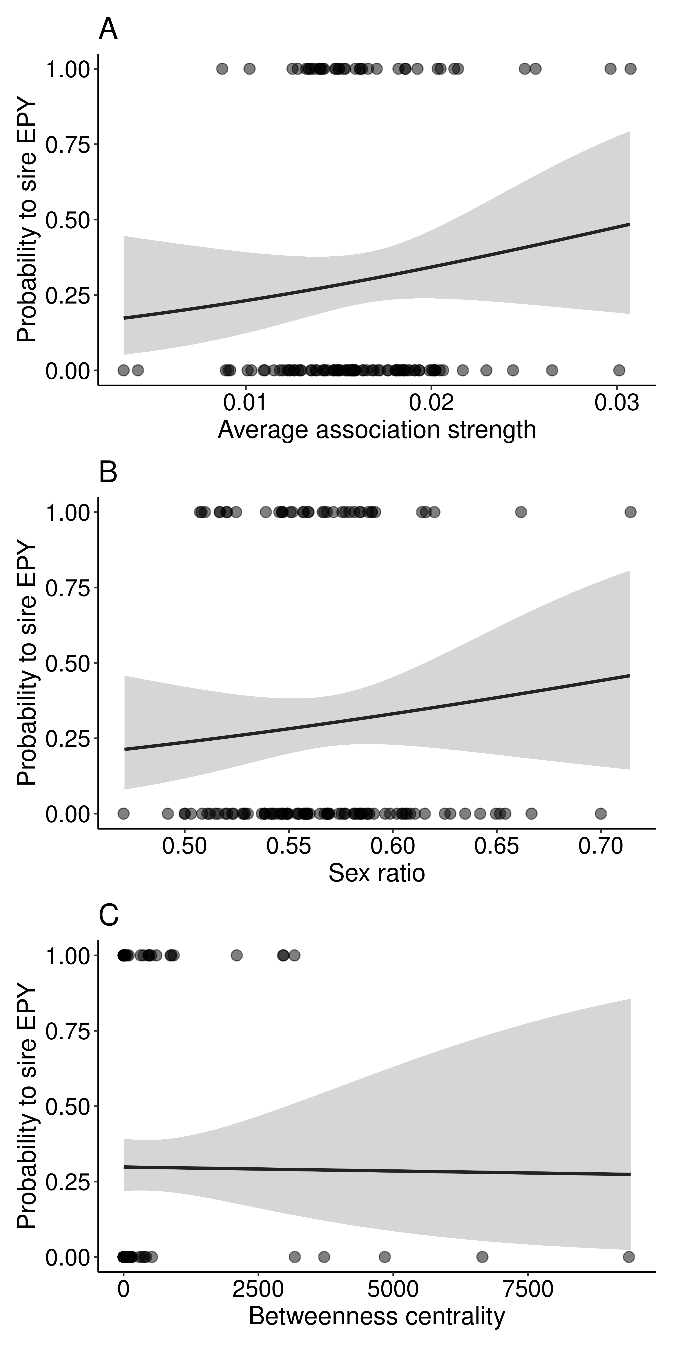


**Figure S6.** Correlates of extra-pair siring success of adult male blue tits. Shown is the predicted probability that a male sired extra-pair young (EPY) in relation to (A) average association strength, (B) sex ratio and (C) betweenness centrality. Dots show the raw data and the grey ribbon shows the 95% confidence interval from the generalized linear model described in the main text while keeping all other independent variables constant at their mean values (standardized effects are shown in Table 2).

**
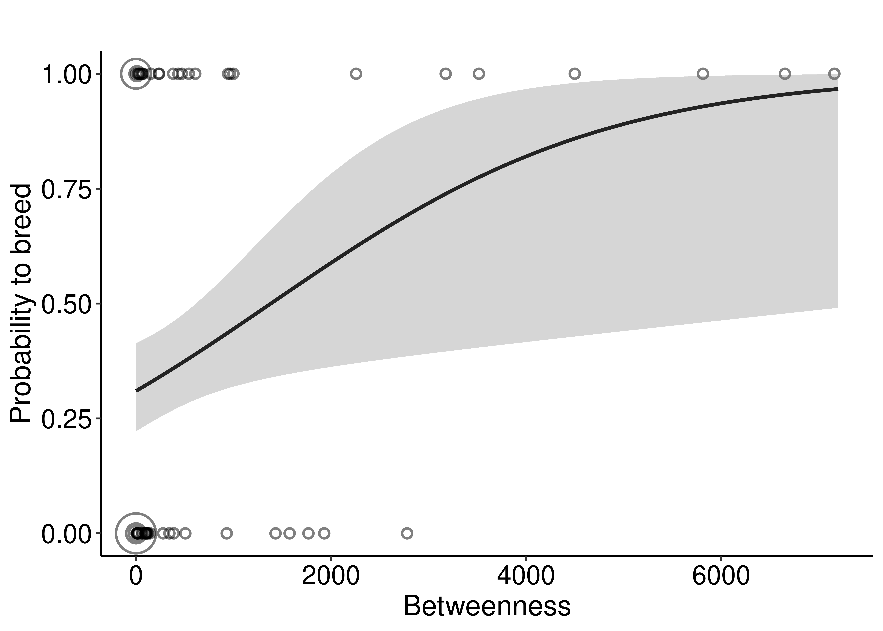
**

**Figure S7**. The predicted probability that a male formed a social pair and bred in relation to the betweenness centrality when excluding one outlier (see Fig. 1A in main text). Dots show the raw data and dot size represents the number of individuals (N=1-30). The grey ribbon shows the 95% confidence interval from the generalized linear model described in the main text while keeping all other independent variables constant at their mean values (standardized effects are shown in Table S4).

**Additional tables**

**Results of the analyses when thresholding the network**

In the analysis on pairing success, results remained unchanged when 5% of weakest edges were removed (Table S1). However, when removing 10% of weakest edges, the effect of betweenness centrality was not statistically significant anymore (Table S5). In the analysis on extra-pair paternity, the effect of the number of female associates was not statistically significant anymore, both when removing 5% or 10% of weakest edges (Table S2, S6). However, when only analyzing breeding males, the effect was still present when removing 5% of weakest edges (Table S3) but not when removing 10% (Table S7). Results for the analyses on the number of breeding neighbors and the proportion of familiar breeding neighbors did not change considerably when removing both 5% and 10% of weakest edges (Table S4, S8).

**5% of lowest edges removed**

**Table S1**. Results of two models examining the effect of the number of opposite-sex associates, the average association strength, the sex ratio, the betweenness centrality, age (yearling vs adult) and arrival date on the likelihood to acquire a social partner and breed for males (N=119) and females (N=93). *P* values inferred from 1000 random permutations are shown in italic (∆: spatially unrestricted null model, *: location-specific null model).

|  | **Males** | |  | **Females** |  |  |
| --- | --- | --- | --- | --- | --- | --- |
| **Fixed effect** | **Estimate ± SE** | **z** | ***P*** | **Estimate ± SE** | **z** | ***P*** |
| Intercept | -0.53 ± 0.22 | -2.47 |  | -0.28 ± 0.24 | -1.15 |  |
| Number of  associates | -0.68 ± 0.52 | -1.31 | *0.18* ^∆^  *0.15 ** | -0.37 ± 0.64 | -0.58 | *0.52* ^∆^  *0.52 ** |
| Average  association strength | -0.06 ± 0.46 | -0.14 | *0.90* ^∆^  *0.88 ** | -0.41 ± 0.53 | -0.77 | *0.44* ^∆^  *0.42 ** |
| Sex ratio | -0.40 ± 0.46 | -0.87 | *0.40* ^∆^  *0.41 ** | 1.15 ± 0.62 | 1.85 | *0.06* ^∆^  *0.07 ** |
| Betweenness  centrality | 1.63 ± 0.58 | 2.80 | *0.01* ^∆^  *0.02 ** | 0.76 ± 0.58 | 1.31 | *0.20* ^∆^  *0.19 ** |
| Age ^a^ | -0.94 ± 0.47 | -2.01 | 0.05 | -1.61 ± 0.60 | -2.68 | 0.01 |
| Arrival date | -1.61 ± 0.49 | -3.28 | 0.001 | -1.06 ± 0.59 | -1.82 | 0.07 |
|  |  |  |  |  |  |  |

^a^ Adults compared to yearlings

**Table S2**. Results of two models examining the effect of the number of opposite-sex associates, the average association strength, the sex ratio and the betweenness centrality on the likelihood to acquire extra-pair young for adult males (N=123) and for females (N=95). *P* values are inferred from 1000 random permutations (∆: spatially unrestricted null model, *: location-specific null model).

|  | **Males** | |  | **Females** |  |  |
| --- | --- | --- | --- | --- | --- | --- |
| **Fixed effect** | **Estimate ± SE** | **z** | ***P*** | **Estimate ± SE** | **z** | ***P*** |
| Intercept | -0.86 ± 0.21 | -4.19 |  | -0.53 ± 0.22 | -2.40 |  |
| Number of  associates | 0.91 ± 0.50 | 1.84 | 0.05 ^∆^  0.06 * | 0.95 ± 0.55 | 1.71 | 0.09 ^∆^  0.07 * |
| Average  association strength | 0.45 ± 0.43 | 1.05 | 0.30 ^∆^  0.33 * | -0.10 ± 0.45 | -0.22 | 0.84 ^∆^  0.83 * |
| Sex ratio | 0.34 ± 0.47 | 0.74 | 0.44 ^∆^  0.54 * | -0.91 ± 0.50 | -1.82 | 0.08 ^∆^  0.07 * |
| Betweenness  centrality | 0.24 ± 0.38 | 0.61 | 0.59 ^∆^  0.56 * | -0.72 ± 0.54 | -1.32 | 0.18 ^∆^  0.18 * |
|  |  |  |  |  |  |  |

**Table S3**. Results of two models examining the effect of the number of opposite-sex associates, the average association strength, the sex ratio and the betweenness centrality on the likelihood to acquire extra-pair young for adult breeding males (N=81). *P* values are inferred from 1000 random permutations (∆: spatially unrestricted null model, *: location-specific null model).

|  | **Males** | |  |
| --- | --- | --- | --- |
| **Fixed effect** | **Estimate ± SE** | **z** | ***P*** |
| Intercept | -0.48 ± 0.24 | -1.99 |  |
| Number of  associates | 1.23 ± 0.58 | 2.12 | 0.03 ^∆^  0.03 * |
| Average  association strength | 0.60 ± 0.53 | 1.14 | 0.26 ^∆^  0.23 * |
| Sex ratio | 0.18 ± 0.53 | 0.34 | 0.71 ^∆^  0.78 * |
| Betweenness  centrality | -0.13 ± 0.49 | -0.27 | 0.81 ^∆^  0.87 * |
|  |  |  |  |

**Table S4.** Results of models examining the effect of the number of female associates during winter on the number and on the proportion of familiar females in the close breeding neighborhood (1^st^ and 2^nd^ order neighbors) of males (N=81). *P* values are inferred from 1000 random permutations (∆: spatially unrestricted null model, *: location-specific null model).

|  | **Number of neighbors** | | | **Proportion of familiar females** | | |
| --- | --- | --- | --- | --- | --- | --- |
|  | **Estimate ± SE** | **z** | ***P*** | **Estimate ± SE** | **z** | ***P*** |
| Intercept | 2.59 ± 0.03 | 84.64 |  | 1.02 ± 0.07 | 14.11 |  |
| Number female associates | 0.09 ± 0.06 | 1.48 | 0.18 ^∆^  0.98 * | 1.19 ± 0.14 | 8.36 | <0.001 ^∆^  <0.001 * |

**10% of lowest edges removed**

**Table S5**. Results of two models examining the effect of the number of opposite-sex associates, the average association strength, the sex ratio, the betweenness centrality, age (yearling vs adult) and arrival date on the likelihood to acquire a social partner and breed for males (N=119) and females (N=93). *P* values inferred from 1000 random permutations are shown in italic (∆: spatially unrestricted null model, *: location-specific null model).

|  | **Males** | |  | **Females** |  |  |
| --- | --- | --- | --- | --- | --- | --- |
| **Fixed effect** | **Estimate ± SE** | **z** | ***P*** | **Estimate ± SE** | **z** | ***P*** |
| Intercept | -0.55 ± 0.21 | -2.62 |  | -0.26 ± 0.24 | -1.06 |  |
| Number of  associates | -0.64 ± 0.53 | -1.20 | *0.23* ^∆^  *0.20 ** | -0.63 ± 0.66 | -0.96 | *0.30* ^∆^  *0.29 ** |
| Average  association strength | 0.51 ± 0.44 | 1.18 | *0.25* ^∆^  *0.24 ** | -0.06 ± 0.53 | -0.11 | *0.91* ^∆^  *0.91 ** |
| Sex ratio | -0.55 ± 0.44 | -1.26 | *0.22* ^∆^  *0.19 ** | 1.43 ± 0.72 | 2.00 | *0.03* ^∆^  *0.04 ** |
| Betweenness  centrality | 0.59 ± 0.50 | 1.18 | *0.26* ^∆^  *0.27 ** | 1.15 ± 0.67 | 1.72 | *0.07* ^∆^  *0.09 ** |
| Age ^a^ | -1.01 ± 0.46 | -2.21 | 0.03 | -1.66 ± 0.62 | -2.69 | 0.01 |
| Arrival date | -1.40 ± 0.47 | -2.97 | 0.003 | -0.93 ± 0.60 | -1.55 | 0.12 |
|  |  |  |  |  |  |  |

^a^ Adults compared to yearlings

**Table S6**. Results of two models examining the effect of the number of opposite-sex associates, the average association strength, the sex ratio and the betweenness centrality on the likelihood to acquire extra-pair young for adult males (N=123) and for females (N=95). *P* values are inferred from 1000 random permutations (∆: spatially unrestricted null model, *: location-specific null model).

|  | **Males** | |  | **Females** |  |  |
| --- | --- | --- | --- | --- | --- | --- |
| **Fixed effect** | **Estimate ± SE** | **z** | ***P*** | **Estimate ± SE** | **z** | ***P*** |
| Intercept | -0.86 ± 0.21 | -4.18 |  | -0.52 ± 0.22 | -239 |  |
| Number of  associates | 0.48 ± 0.52 | 0.92 | 0.32 ^∆^  0.44 * | 0.76 ± 0.59 | 1.28 | 0.08 ^∆^  0.07 * |
| Average  association strength | 0.74 ± 0.42 | 1.77 | 0.08 ^∆^  0.09 * | -0.07 ± 0.46 | -0.15 | 0.96 ^∆^  0.96 * |
| Sex ratio | -0.02 ± 0.44 | -0.05 | 0.96 ^∆^  0.97 * | 1.13 ± 0.56 | 2.01 | 0.07 ^∆^  0.08 * |
| Betweenness  centrality | 0.41 ± 0.45 | 0.92 | 0.43 ^∆^  0.43 * | -0.01 ± 0.51 | -1.47 | 0.10 ^∆^  0.10 * |
|  |  |  |  |  |  |  |

**Table S7**. Results of two models examining the effect of the number of opposite-sex associates, the average association strength, the sex ratio and the betweenness centrality on the likelihood to acquire extra-pair young for adult breeding males (N=81). *P* values are inferred from 1000 random permutations (∆: spatially unrestricted null model, *: location-specific null model).

|  | **Males** | |  |
| --- | --- | --- | --- |
| **Fixed effect** | **Estimate ± SE** | **z** | ***P*** |
| Intercept | -0.86 ± 0.21 | -4.18 |  |
| Number of  associates | 0.48 ± 0.53 | 0.92 | 0.36 ^∆^  0.45 * |
| Average  association strength | 0.74 ± 0.42 | 1.77 | 0.07 ^∆^  0.11 * |
| Sex ratio | -0.02 ± 0.44 | -0.05 | 0.96 ^∆^  0.97 * |
| Betweenness  centrality | 0.41 ± 0.45 | 0.92 | 0.41 ^∆^  0.42 * |
|  |  |  |  |

**Table S8.** Results of models examining the effect of the number of female associates during winter on the number and on the proportion of familiar females in the close breeding neighborhood (1^st^ and 2^nd^ order neighbors) of males (N=81). *P* values are inferred from 1000 random permutations (∆: spatially unrestricted null model, *: location-specific null model).

|  | **Number of neighbors** | | | **Proportion of familiar females** | | |
| --- | --- | --- | --- | --- | --- | --- |
|  | **Estimate ± SE** | **z** | ***P*** | **Estimate ± SE** | **z** | ***P*** |
| Intercept | 2.59 ± 0.03 | 84.65 |  | 1.02 ± 0.07 | 14.12 |  |
| Number female associates | 0.09 ± 0.06 | 1.42 | 0.15 ^∆^  0.99 * | **1.20 ± 0.14** | **8.42** | **<0.001** ^∆^  **<0.001** * |

**Table S9.** Microsatellite loci for blue tits. Primer sequences include information on fluorescence labels used. *C* refers to the primer concentration in multiplex primer mix. Size range and number of alleles refer to 2018 data (*n*=1696; Phtr3 from 2017, *n*=1905).

| **Locus** | **Accession no.** | **published in** | **Primer sequences (5’ - 3’)** | ***C* (μM)** | **Multiplex Mix** | **Size range (bp)** | **number of alleles** |
| --- | --- | --- | --- | --- | --- | --- | --- |
| **ADCYAP1_bm** | FJ464427 | Steinmeyer et al (2009), supplement | VIC-GATGTGAGTAACCAGCCACT  ATAACACAGGAGCGGTGA | 0,2 μM | 2 | 160 - 172 | 10 |
| **ClkpolyQ** | AY338423-28 | Johnsen et al (2007) | 6FAM-TTTTCTCAAGGTCAGCAGCTTGT  CTGTAGGAACTGTTGYGGKTGCTG | 0,36 μM | 4 | 266 - 283 | 7 |
| **Mcµ4** | U82388 | Double et al. (1997) | PET-ATAAGATGACTAAGGTCTCTGGTG  TAGCAATTGTCTATCATGGTTTG | 1,1 μM | 2 | 156 - 194 | 19 |
| **PAT MP 2-43** | AM056063 | Otter et el. (1998) | 6FAM- ACAGGTAGTCAGAAATGGAAAG  GTATCCAGAGTCTTTGCTGATG | 0,24 μM | 4 | 125 - 155 | 8 |
| **Pca3** | AJ279805 | Dawson et al. (2000) | PET-GGTGTTTGTGAGCCGGGG  TGTTACAACCAAAGCGGTCATTTG | 0,8 μM | 1 | 154 - 234 | 43 |
| **Pca4** | AJ279806 | Dawson et al. (2000) | NED-AATGTCTTACAGGCAAAGTCCCCA  AACTTGAAGCTTCTGGCCTGAATG | 0,42 μM | 4 | 149 - 201 | 18 |
| **Pca7** | AJ279809 | Dawson et al. (2000) | 6FAM-TGAGCATCGTAGCCCAGCAG  GGTTCAGGACACCTGCACAATG | 0,25 μM | 1 | 105 - 141 | 18 |
| **Pca8** | AJ279810 | Dawson et al. (2000) | NED-ACTTCTGAAACAAAGATGAAATCA  TGCCATCAGTGTCAAACCTG | 0,48 μM | 1 | 255 - 401 | 73 |
| **Pca9** | AJ279811 | Dawson et al. (2000) | VIC-ACCCACTGTCCAGAGCAGGG  AGGACTGCAGCAGTTTGTGGG | 0,3 μM | 3 | 111 - 135 | 13 |
| **Phtr3 ¹** | AM056070 | Fridolfsson et al. (1997) | NED-ATTTGCATCCAGTCTTCAGTAATT  CTCAAAGAAGTGCATAGAGATTTCAT | 1,4 μM | 2 **¹** | 118 - 148 **¹** | 16 **¹** |
| **PK11** | AF041465 | Tanner,S.M., Richner,H. and Schuemperli,D.; unpublished | PET-CTTTAAGAATTCAAATACAGAGTAGG  GTTTTCTCCTTTCTACACTGAGG | 0,54 μM | 4 | 63 - 97 | 14 |
| **PK12** | AF041466 | Tanner,S.M., Richner,H. and Schuemperli,D.; unpublished | VIC-CCTCCTGCAGTTGCCTCCCG  CGTGGCCATGTTTATAGCCTGGCACTAAGAAC | 1,14 μM | 4 | 168 - 226 | 27 |
| **PmaTAGAn71 ¹** | AY260537 | Saladin et el. (2003) | NED-TCAGCCTCCAAGGAAAACAG  GCATAAGCAACACCATGCAG | 0,3 μM | 2 **¹** | 190 - 310 **¹** | 29 **¹** |
| **POCC1** | U59113 | Bensch et al. (1997) | 6FAM- TTCTGTGCTGCAATCACACA  GCTTCCAGCACCACTTCAAT | 0,8 μM | 3 | 219 - 255 | 25 |
| **POCC6** | U59117 | Bensch et al. (1997) | VIC-TCACCCTCAAAAACACACACA  ACTTCTCTCTGAAAAGGGGAGC | 0,25 μM | 1 | 195 - 253 | 28 |
| **P2/P8** | AF006659-62 | Griffiths et al. (1998) | 6FAM-CTCCCAAGGA TGAGRAAYTG  TCTGCATCGC TAAATCCTTT | 0,3 μM | 2 | 319, 383 | 2 |

¹ Phtr3 was replaced by PmaTAGAn71 from 2018 onwards.

**Table S10.** Spearman correlation coefficients (rho) of the social network metrics (i.e. number of opposite-sex associates, the average association strength, the sex ratio, the betweenness centrality) and arrival date. Values below the shaded diagonal cells are from the female dataset used in the analysis on pairing success, values above from the male dataset.

|  | **Number of**  **associates** | **Average**  **association strength** | **Sex ratio** | **Betweenness**  **centrality** | **Arrival date** |
| --- | --- | --- | --- | --- | --- |
| **Number of**  **associates** |  | 0.06 | -0.26 | 0.45 | -0.23 |
| **Average**  **association strength** | 0.37 |  | -0.25 | 0.17 | -0.16 |
| **Sex ratio** | -0.03 | -0.02 |  | -0.07 | 0.01 |
| **Betweenness**  **centrality** | 0.53 | 0.47 | -0.001 |  | -0.21 |
| **Arrival date** | -0.49 | -0.29 | -0.08 | -0.37 |  |

**Table S11.** Spearman correlation coefficients (rho) of the social network metrics, i.e. number of opposite-sex associates, the average association strength, the sex ratio and the betweenness centrality. Values below the shaded diagonal cells are from the female dataset used in the analysis on extra-pair paternity, values above for the male dataset.

|  | **Number of**  **associates** | **Average**  **association strength** | **Sex ratio** | **Betweenness**  **centrality** |
| --- | --- | --- | --- | --- |
| **Number of associates** |  | 0.08 | -0.32 | 0.43 |
| **Average association strength** | 0.31 |  | -0.14 | 0.28 |
| **Sex ratio** | -0.13 | 0.15 |  | 0.07 |
| **Betweenness Centrality** | 0.53 | 0.13 | -0.05 |  |

**Table S12.** Results of two models examining the effect of the number of opposite-sex associates, the average association strength, the sex ratio, the betweenness centrality and arrival date on the likelihood to acquire a social partner and breed for yearling males (N=75) and yearling females (N=67). Significant *P* values are shown in bold. *P* values inferred from 1000 random permutations are shown in italic (∆: spatially unrestricted null model, *: location-specific null model).

|  | **Yearling males** | |  | **Yearling females** | |  |
| --- | --- | --- | --- | --- | --- | --- |
| **Fixed effect** | **Estimate ± SE** | **Z** | ***P*** | **Estimate ± SE** | **z** | ***P*** |
| Intercept | -0.24 ± 0.28 | -0.88 |  | 0.19 ± 0.26 | 0.73 |  |
| Number of  associates | 0.26 ± 0.66 | 0.38 | *0.68* ^∆^  *0.68 ** | -0.39 ± 0.69 | -0.56 | *0.58* ^∆^  *0.51 ** |
| Average  association strength | 1.09 ± 0.65 | 1.67 | *0.08* ^∆^  *0.07 ** | 0.05 ± 0.58 | 0.09 | *0.95* ^∆^  *0.93 ** |
| Sex ratio | -0.11 ± 0.67 | -0.16 | *0.86* ^∆^  *0.93 ** | 0.62 ± 0.60 | 1.04 | *0.31* ^∆^  *0.34 ** |
| Betweenness  centrality | 1.06 ± 0.90 | 1.18 | *0.19* ^∆^  *0.32 ** | 0.46 ± 0.66 | 0.70 | *0.49* ^∆^  *0.45 ** |
| Arrival date | **-1.69 ± 0.64** | **-2.66** | **0.008** | **-1.43 ± 0.66** | **-2.18** | **0.03** |
|  |  |  |  |  |  |  |

**Table S13**. Results of two models examining the effect of the number of opposite-sex associates, the average association strength, the sex ratio and the betweenness centrality on the likelihood to acquire extra-pair young for adult males that were also detected breeding in our study site (N=81). *P* values are inferred from 1000 random permutations (∆: spatially unrestricted null model, *: location-specific null model).

| **Fixed effect** | **Estimate ± SE** | **z** | ***P*** |
| --- | --- | --- | --- |
| Intercept | -0.48 ± 0.24 | -1.20 |  |
| Number of  associates | **1.25 ± 0.57** | **2.19** | **0.02** ^∆^  **0.02 *** |
| Average  association strength | 0.64 ± 0.53 | 1.22 | 0.22 ^∆^  0.23 * |
| Sex ratio | 0.23 ± 0.53 | 0.43 | 0.66 ^∆^  0.74 * |
| Betweenness  centrality | -0.40 ± 0.53 | -0.75 | 0.47 ^∆^  0.54 * |
|  |  |  |  |

**Table S14**. Results of the models examining the effect of the number of opposite-sex associates, the average association strength, the sex ratio, the betweenness centrality, age and the arrival time on the likelihood to acquire a mate in males when excluding one outlier (N = 118). Significant *P* values are shown in bold. *P* values inferred from 1000 random permutations are shown in italic (∆: spatially unrestricted null model, *: location-specific null model).

| **Fixed effect** | **Estimate ± SE** | **z** | ***P*** |
| --- | --- | --- | --- |
| Intercept | -0.54 ± 0.22 | -2.50 |  |
| Number of  associates | -0.37 ± 0.50 | -0.74 | *0.45* ^∆^  *0.40 ** |
| Average  association strength | 0.28 ± 0.48 | 0.58 | *0.54* ^∆^  *0.53 ** |
| Sex ratio | -0.20 ± 0.47 | -0.43 | *0.67* ^∆^  *0.64 ** |
| Betweenness  centrality | **1.43 ± 0.62** | **2.31** | ***0.03*** ^∆^  ***0.02 **** |
| Age ^a^ | **-1.00 ± 0.47** | **-2.13** | **0.03** |
| Arrival time | **-1.49 ± 0.49** | **-3.04** | **0.002** |
|  |  |  |  |

^a^ Adults compared to yearlings

**References**

Dawson DA, Hanotte O, Greig C, Stewart IRK, Burke T (2000) Polymorphic microsatellites in the Blue Tit Parus caeruleus and their cross-species utility in 20 songbird families. Molecular Ecology, 9, 1941–1944.

Fridolfsson AK, Gyllensten UB, Jakobsson S (1997) Microsatellite markers for paternity testing in the willow warbler Phylloscopus trochilus: high frequency of extra-pair young in an island population. Hereditas, 126, 127–132.

Johnsen A, Fidler AE, Kuhn S et al. (2007) Avian Clock gene polymorphism: evidence for a latitudinal cline in allele frequencies. Molecular Ecology, 16, 4867–4880.

Griffiths, R., M. C. Double, K. Orr, and R. J. G. Dawson. 1998. A DNA test to sex most birds. Molecular Ecology 7:1071–1075.

Steinmeyer C, Mueller JC, Kempenaers B (2009) Search for informative polymorphisms in candidate genes: clock genes and circadian behaviour in blue tits. Genetica 136:109–117

Saladin V, Bonfils D, Binz T, Richner H (2003) Isolation and characterization of 16 microsatellite loci in the Great Tit Parus major. Molecular Ecology Notes, 3, 520–522.

Bensch S, Price T, Kohn J (1997) Isolation and characterization of microsatellite loci in a Phylloscopus warbler. Molecular Ecology, 6, 91–92.

Otter,K., Ratcliffe,L., Michaud,D. and Boag,P.T. (1998) Do female black-capped chickadees prefer high-ranking males as extra-pair partners. Behav. Ecol. Sociobiol. 43, 25-36

Psorakis I, Roberts SJ, Rezek I, Sheldon BC. (2012). Inferring social network structure in ecological systems from spatio-temporal data streams. J R Soc Interface. 9:3055–3066.

Tanner, S. M., Richner, H., & Schuemmperli, D. (1995). Microsatellite-DNA-fingerprinting in blue tits (Parus caeruleus) by the polymerase chain reaction (Doctoral dissertation, Diploma thesis, University of Bern, Germany).
